# Measurement and monitoring of electrocardiogram belt tension in premature infants for assessment of respiratory function

**DOI:** 10.1186/1475-925X-6-13

**Published:** 2007-04-19

**Authors:** Edward J Ciaccio, Mark Hiatt, Thomas Hegyi, Gary M Drzewiecki

**Affiliations:** 1 Department of Pharmacology, Columbia University, New York, USA; 2 Department of Biomedical Engineering, Columbia University, New York, USA; 3 Department of Pediatrics, Saint Peter's Medical Center, New Brunswick, USA; 4 Department of Pediatrics, University of Medicine and Dentistry of New Jersey – Robert Wood Johnson Medical School, New Brunswick, USA; 5 Department of Biomedical Engineering, Rutgers University, New Brunswick, USA

## Abstract

**Background:**

Monitoring of the electrocardiogram (ECG) in premature infants with conventional adhesive-backed electrodes can harm their sensitive skin. Use of an electrode belt prevents skin irritation, but the effect of belt pressure on respiratory function is unknown. A strain gauge sensor is described which measures applied belt tension.

**Method:**

The device frame was comprised of an aluminum housing and slide to minimize the device weight. Velcro tabs connected housing and slide to opposite tabs located at the electrode belt ends. The slide was connected to a leaf spring, to which were bonded two piezoresistive transducers in a half-bridge circuit configuration. The device was tested for linearity and calibrated. The effect on infant respiratory function of constant belt tension in the normal range (30 g–90 g) was determined.

**Results:**

The mechanical response to a step input was second order (f_n _= 401 Hz, ζ = 0.08). The relationship between applied tension and output voltage was linear in the range 25–225 gm of applied tension (r^2 ^= 0.99). Measured device sensitivity was 2.18 mV/gm tension using a 5 V bridge excitation voltage. When belt tension was increased in the normal range from 30 gm to 90 gm, there was no significant change in heart rate and most respiratory functions during monitoring. At an intermediate level of tension of 50 gm, pulmonary resistance and work of breathing significantly decreased.

**Conclusion:**

The mechanical and electrical design of a device for monitoring electrocardiogram electrode belt tension is described. Within the typical range of application tension, cardiovascular and respiratory function are not substantially negatively affected by electrode belt force.

## Background

Electrode belts with standard leads are commonly used devices for monitoring heart rate [1-4] and its variability [5], to assess physical activity [6], for instrumentation of ergometers used in sports medicine studies [7], aerospace medicine studies [8], for electrocardiogram (ECG) monitoring and defibrillation [9], as well as for electrical impedance tomography [10]. Similarly, arrays of many electrodes fitted within electrode vests are used to monitor the spatial distribution of heart signals, and can be either strapped to the chest [11] or held in place by a pneumatic mechanism [12]. The importance heart monitoring with electrode arrays to indicate regions of bioelectric abnormality where cardiac arrhythmias can originate is firmly established [13-16].

Premature infants confined to the neonatal intensive care unit (ICU) are routinely monitored with electrodes to obtain a record of the electrocardiogram (ECG). Application of standard electrodes to a premature infant's skin in the required locations can be tedious and time consuming [17]. Furthermore, the skin of premature infants is delicate and sensitive, and application and removal of contact tape can injure the patient. To overcome this problem, ECG electrodes can be mounted on a rubberized belt and positioned at the required location for recording by wrapping the device about the subject's torso [17]. Electrode belts can be used for both short-term and long-term ECG monitoring [18]. By use of a belt rather than contact tape to position individual electrodes to the chest, skin irritation is avoided. There is also ease of electrode application and removal. However, premature infants typically suffer from diminished respiratory function, which may be complicated by an inappropriately large level of belt tension.

Very premature infants, presently born as young as 20 weeks gestational age [19], may be as light as 400–500 grams [20], and have chests approximately 5 cm in diameter. To monitor electrode belt tension would require construction of a miniaturized device that could be attached between the tabs that are used to fasten the belt. Herein we describe the design and implementation of such a device, which is then used to quantify electrode belt tension in a premature infant. We compare monitored respiratory parameters to the measured tension levels that are applied in the ICU.

## Methods

To measure tension applied normal to the axis of the electrode belt, the device width should be significantly less than chest diameter. Further, to prevent application of a vertical torque during measurement, the frame thickness must be minimized. A stiff, lightweight frame material is needed to prevent bending of the frame and to minimize the weight on the infant's chest. The device must be electrically isolated from the infant's skin. The mechanical and electrical construction of the device took into account these considerations.

### Device housing and transducers

**Figure 1:**
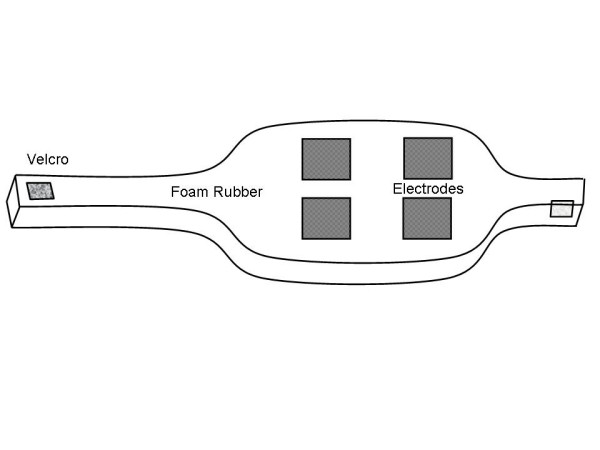
Electrode belt detail. The device is composed of foam rubber with embedded electrodes. Velcro tabs are located at the ends to fasten it in place when it is wrapped around a patient.

The electrode belt allows correct positioning of ECG electrodes on the patient without the need of adhesive. The electrode belt that was used for this purpose consists of conductive carbon electrodes that are mounted on foam rubber backing, and fitted snugly about an infant's chest by means of Velcro fasteners (InfanTrode, Survival Technology Inc., Rockville MD). An illustration is shown in Fig. 1. The belt is 28 cm in length and composed of foam rubber, with flexible plastic portions which contain the electrodes.

**Figure 2:**
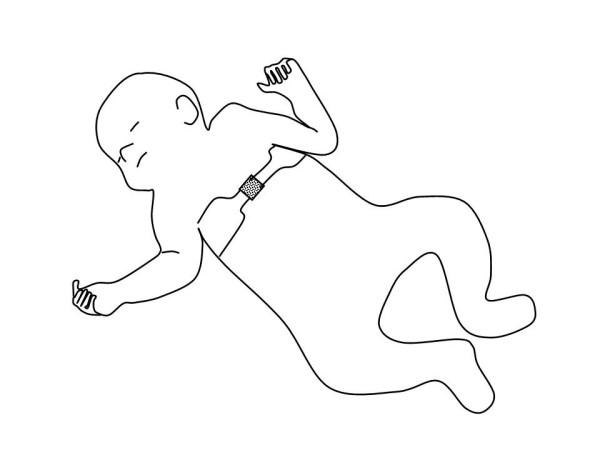
Schematic showing the positioning of the electrode belt about the infant's chest. Square at center denotes the position of the tension gauge device.

**Figure 3:**
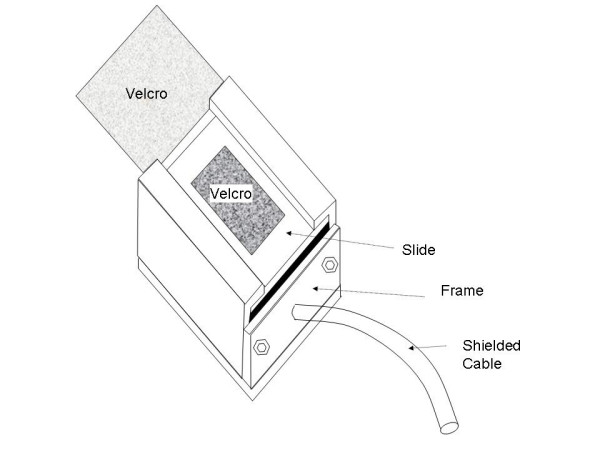
Tension gauge device. The location of Velcro tabs which adjoin with corresponding tabs on the electrode belt are shown. One Velcro tab is affixed to the slide (top of the device as shown) while the other is affixed to the device housing. Machine screws are used to attach the separate components of the device frame. The piezoresistive sensors are in continuity with the signal conditioning circuit via the cable shown.

The tension gauge instrument that was devised is shown schematically in Fig. 2 as being attached to the ends of the electrode belt (denoted as square at infant's belly). The mechanical portions of the device are shown in Fig. 3. They consist of an aluminum frame and slide (mass = 36 and 2.1 grams, respectively), a leaf spring (stainless steel, Young's Modulus of Elasticity E = 29.5E6 lb/in^2^), pin (identical material as frame), and Velcro tabs which connect with the ends of the electrode belt.

**Figure 4:**
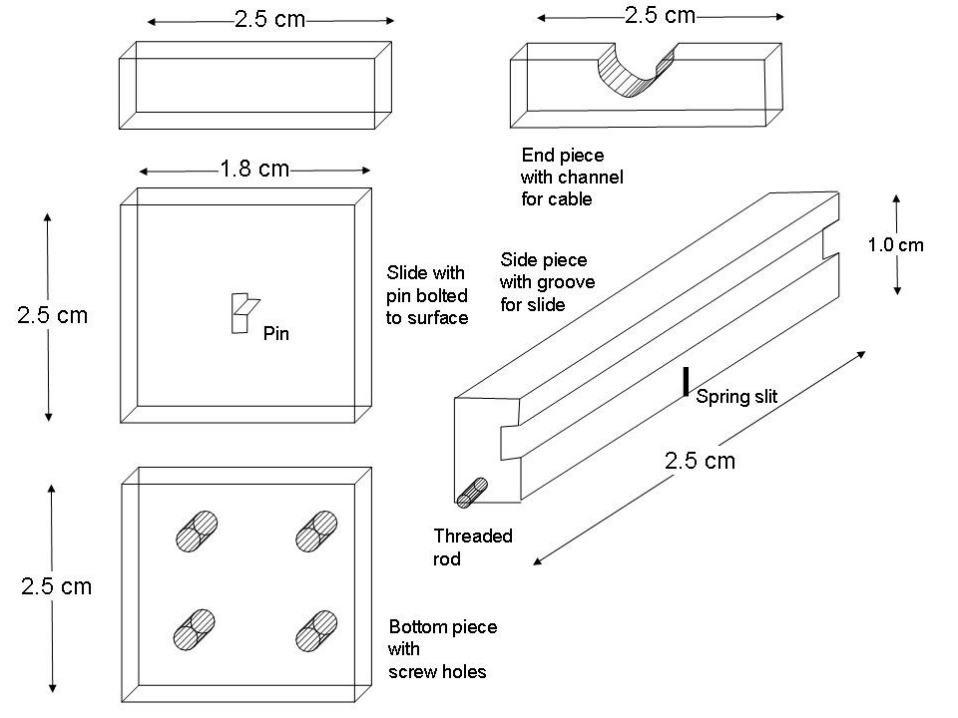
The separate components of the frame. A pin is attached to the slide. The slide moves along grooves cut into side pieces. The leaf spring bends as the pin to which it is attached moves with the slide as belt tension changes.

The individual mechanical components of the device are shown disassembled in Fig. 4. Each piece interconnects, and the assembled device, when grounded, provides shielding from electrical noise through the Faraday cage principle. A Faraday cage is an enclosure formed by conducting material to block external electrical fields. The separate portions of the frame shown in Fig. 4 were joined by means of steel machine screws. Velcro fasteners, applied to the frame with their original adhesive backing, provided connection with opposite Velcro tabs on the electrode belt. Disposable cotton material was affixed by means of double-sided masking tape to the portion of the instrument's surface that was to be in contact with the infant's chest. The masking tape enabled replacement of cotton, and the instrument was cleaned with alcohol between monitoring periods.

**Figure 5:**
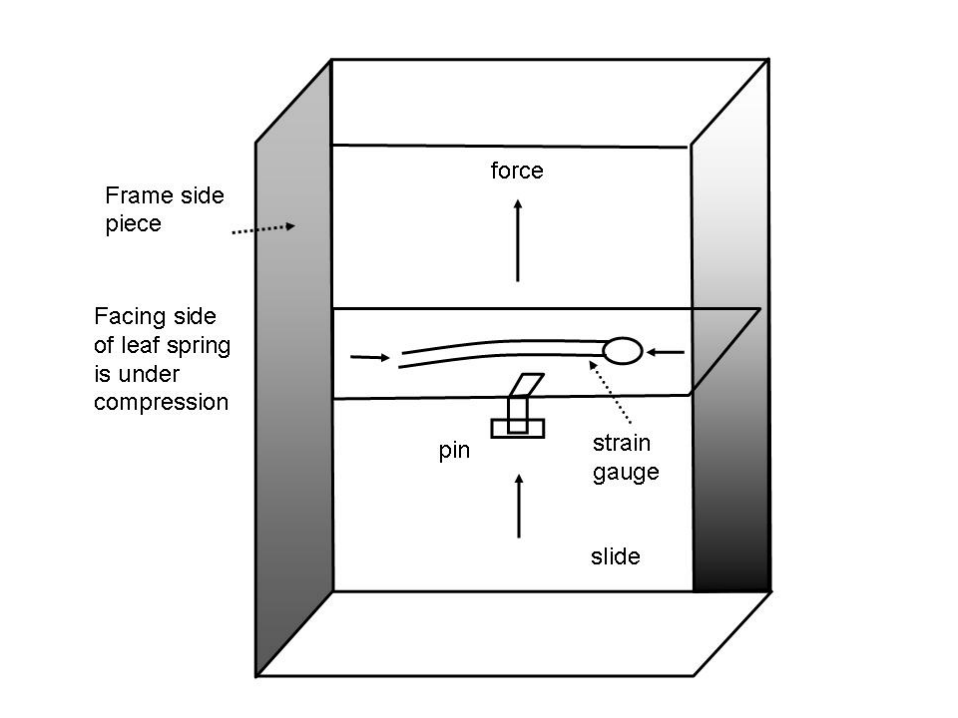
Close-up of slide-pin-leaf spring mechanism with piezoresistive strain gauges attached. The strain gauges are bonded to either side of the leaf spring with an insulating epoxy. Their ends are connected to a cable which is in continuity with the signal conditioning circuit.

The belt tension was monitored by means of two piezoresistive semiconductor elements (Kulite Semiconductor Products, Ridgefield, NJ) which were bonded to either side of a leaf spring. The bonding epoxy (Master Bond, Inc., Teaneck, NJ) was selected for its properties of low application viscosity (to minimize the epoxy layer thickness, and therefore the curing time) and low viscoelasticity (to minimize the tendency for creep deformation to occur during bending). The piezoresistive transducers were used to measure the tension developed in the electrode belt when it is fitted about the patient during intensive care monitoring. The configuration within the device housing is shown in Fig. 5. The leaf spring was wedged into slots cut into the frame by means of steel shims. Epoxy was applied at the wedged areas to enhance stability of the mounting. A pin, bolted to the slide, and tightly fitted around the spring's thickness dimension, provides connection between slide and spring. The slide is the dynamic portion of the instrument's frame which moves the leaf spring. As belt tension increases, the force exerted on the slide bends the leaf spring and the bonded strain gauges. Gold leads from each piezoresistive element were connected with an electrically conductive silver epoxy (Epoxy Technology Inc, Billerica, MA) to individual leads from a four-strand, miniature shielded cable (Alpha Products Corp., Elizabeth, NJ). The conductive epoxy was cured for 90 minutes at 80°C in accord with the manufacturer specifications, and taking into account the recommended high temperature limit of the strain gauge (200°C) and cable (150°C). The cable exited the device through a conduit drilled in the frame and provided communication with the signal conditioning electronics. These electronics were located within a specially constructed aluminum case that was grounded to shield the circuit from line frequency and enhance portability.

### Signal conditioning electronics

**Figure 6:**
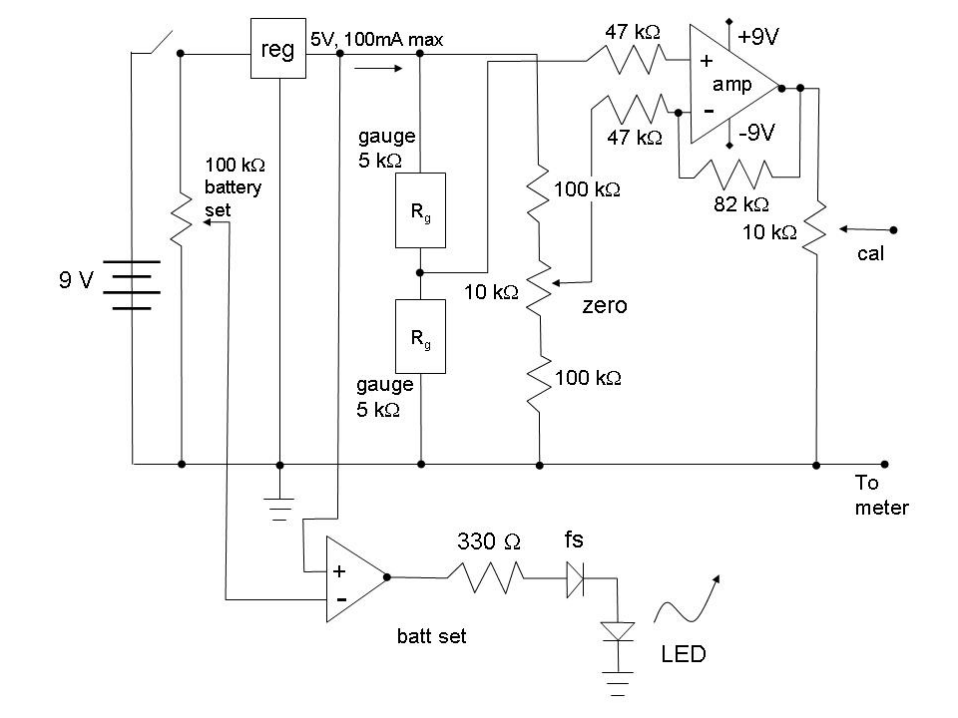
Signal conditioning circuit. The device is powered by nine volt batteries. The regulator adjusts the excitation voltage to the bridge to 5 V. The half-bridge consists of the variable piezoresistances along one arm and the adjusting resistances on the other arm that are used to balance the output when no tension is applied. The output signal is amplified and is calibrated so that 1 gm tension = 1 mV; thus a millivoltmeter can be used for 1:1 reading of grams of tension sensed by the device. The battery low component of the circuit is given at bottom.

The signal conditioning elements are drawn in Fig. 6. The circuit is powered by two nine volt batteries that are configured to provide a two-sided supply. Features of the circuit include:

• ± 9 volt battery power supply

• 5 V, low power voltage regulator (provides stable excitation voltage)

• LED; on asserts battery low condition

• Wheatstone bridge zero adjust

• calibration adjust to relate millivolts to grams of applied tension

The current drawn by the resistive circuit elements and voltage regulator provide an estimate of battery life according to the following equation:

I_circuit _= I_voltreg _+ I_amp _+ I_activearm _+ I_zero _+ I_cal _+ I_batset _

7.9 ma = 2.6 ma +3.8 ma + 0.7 ma + 0.7 ma + 0.1 ma + 0.1 ma

The current drawn by the entire circuit I_circuit _in Eq. 1 is a summation of the current I_volt reg _drawn by the voltage regulator (reg), the current I_amp _of the amplifier (amp), the current I_active arm _through the active arm of the Wheatstone bridge (though 2R_g_), the current I_zero _of the zeroing element (zero), the current I_cal _through the calibration component (cal), and the current I_bat set _through the battery set circuitry (batt set). The lifetime of the power supply to maintain the ≥ 6.2 volts required by voltage regulator for constant 5.0 V output can be calculated as:

0.450 ma·hours/7.9 ma ≈ 55 hours

where the battery life of 0.450 ma·hours was obtained from the battery specifications. When there is less than 6.2 V at the regulator input (battery low condition), the excitation voltage to the Wheatstone bridge is no longer maintained at 5 volts, thereby introducing measurement error. The light emitting diode (LED) alerts the user to a battery low condition. LED off draws approximately no current, however LED on (battery low) was measured to draw an additional 10.2 ma (the forward current requirement). After balancing, if no tension is exerted on the leaf spring, the output will register at zero millivolts. As tension is exerted, a difference in resistance at each strain gauge is created that is translated into a potential difference across the half-active bridge (Fig. 6). The differential bridge voltage is reduced via the calibration adjustment to enable a 100 mV output to correspond to 100 grams of tension (100 g·980 cm/sec^2 ^= 0.98 Newtons).

The piezoresistive transducing elements (Kulite Semiconductor Products, Basingstoke, England) have a stated strain gauge constant of G = 175 ± 5% at 75°C, and resistance R = 5000Ω ± 1%. The maximum strain for linearity for these elements is 1000 με (units are microstrain, which is strain expressed as parts per million). For a rectangular beam with centrally applied force, we calculated the beam thickness needed to maintain linearity. The maximum allowable strain before irreversible deformation can be calculated as follows. The strain is given by [21]:

S = ΔL/L = (3 • F • L)/(4 E b h^2^)

where F is the applied force in Newtons, L is the beam length, b is the beam width (or height), h is the beam thickness, and E is Young's modulus of elasticity. Rearranging the previous equation, the maximum allowable force is:

F_max _= S_max _• (4 E b h^2^)/(3 • L)

where the chosen values of parameters L and b were designed to minimize the size and weight of the device, and h was adjusted according to the required maximum force F_max_. From the initial experience with placing the electrode belt about the patient, normally applied levels of belt tension by clinical personnel in the ICU ranged from approximately 30 g–90 g. A maximum force that would be encountered about the belt due to patient breathing and motion might therefore be about 100 g of tension. To provide for maximum sensitivity with a margin of safety double the maximum estimated force to be encountered, we supposed that the maximum belt tension would be 200 g, and:

F_max _= 200 g • 9.8 N/1000 g = 1.96 N (5)

Rearranging Eq. 4:

h = [(3 • L • F_max_)/(4 • E • b • S_max_)]^1/2 ^= [(3 • 1.56 cm • 1.96 N)/(4 • 20.3E6 N/cm^2 ^• 0.244 cm • 0.001 cm/cm) ]^1/2 ^= 0.021 cm = 0.21 mm

which is the required thickness of the steel leaf spring for maximum sensitivity.

### Transducer sensitivity

For a half-active bridge, the output is [22]:

V_out _= V_in _• ΔR_g_/2R_g _

where R_g _is the strain gauge resistance, and the sensitivity is given in units of differential output voltage V_out _per gram of tension per bridge input voltage V_in_. The relationship between the gauge factor, strain, and resistive changes for a half-active bridge is [22]:

ΔR/R = G • ΔL/L = 175 • 5.25 με = 9.19 × 10^-4 ^

where G and ΔL/L are properties of the particular strain gage that was used, and με are units of microstrain. For 1 gram (gm) of tension, the sensitivity is V_out_/1 gm tension/5 V input excitation:

sensitivity = (5.0 V • 9.19 E-4/2)/gm tension/5 V_ex _= 2.30 mV/gm tension/5 V_ex_

where T = tension and V_ex _is the excitation voltage of the bridge. Therefore, without adjusting the gain, a 100 gm input tension would be expected to generate a 230 mV output. The unit was tested for electrostatic discharge and electrical isolation prior to connecting it to the EKG belt on actual patients.

### Clinical protocol

Clinical monitoring was done at Saint Peter's Medical Center in New Brunswick NJ. An infant's candidacy for monitoring required that an electrode belt was already being used for ECG recording, the subject was sleeping in the supine position, and that the infant's normal schedule of care was not interrupted. The patient's physician applied the electrode belt with tension monitor in place. A technician and an engineer assisted the physician with the device, data processing equipment, and readout. Tension was applied at 30 gm as measured by the tension gauge, and the infant's position was adjusted to allow approximately even distribution of the applied belt tension. After waiting two minutes for the infant and the device to adjust, respiratory function was sampled using a pneumotach (MAS Inc, Hatfield PA). The functions that were measured were: respiratory frequency, tidal volume, and work of breathing. The procedure was then repeated at 50 gm and 90 gm of tension. The mean and standard deviation of each measurement taken at 20 minute intervals over a 2 hour period were tabulated.

## Results

### Bench tests

**Figure 7:**
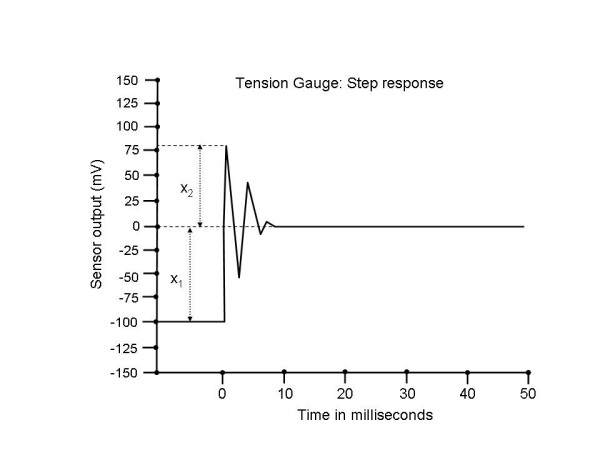
The step response to the tension gauge is a second order function. The resonant frequency and damping coefficient can be mathematically computed from this response (see text).

The mechanical and electrical response of the tension gauge were tested following construction of the device. The mechanical step response was determined by anchoring a taut elastic band to the slide, followed by quick release. The resulting curve indicates an underdamped second order mechanical response (Fig. 7). The damping coefficient, ζ, and the resonant frequency, f_n_, can be respectively determined as follows [23]:

ζ = [(ln(x_2_/x_1_))^2^/(π^2 ^+ (ln(x_2_/x_1_))^2^]^1/2 ^

f_n _= f_d_/(1 - ζ^2^)^1/2 ^

where x_1 _and x_2 _are the step input and first maximum, respectively, measured x_2_/x_1 _from Fig. 7 was 0.78, and T_d_, the time from first to second maximum, was 2.5 ms from Fig. 7, and f_d _= 1/T_d _= 400 Hz. Thus from Eq. 10, ζ = .08 and f_n _= 401 Hz. The device was designed to measure the DC level of tension and low frequency components, which are well below the resonant frequency of this device.

Force was then applied to different portions of the frame with varying amplitudes and directions, while output of the tension gauge was tabulated. A 1–3 mV output was observed upon compression of the sensor with approximately 100 Newtons of force applied, which was beyond any force the housing would be expected to encounter. With light pressure the output remained at 0 mV.

**Figure 8:**
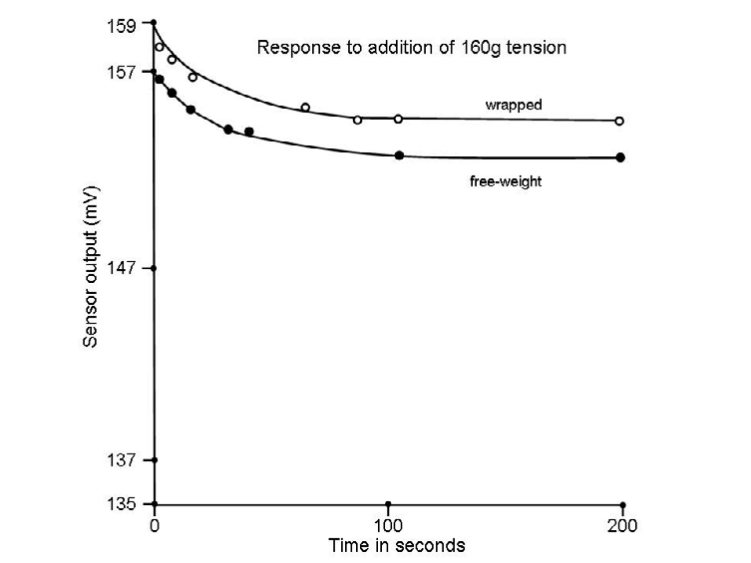
Creep effect when a level tension is applied to the device by either hanging weights or wrapping the device about a cylinder. The amount of creep is approximately 10% and it occurs over several minutes' time.

The tension gauge frame was then clamped to the edge of a desk top, while weights of known value were attached to the slide, via the Velcro communication, and allowed to swing freely to study the effect of constant stress on the measurement output [24]. The weights were released by hand as rapidly as possible without bouncing. The sensor output was measured at 7 intervals over a 200s period. Some creep was noted in the output (Fig. 8, solid circles), which is attributable to the viscoelastic properties of the epoxy. Any viscoelastic effects by the electrode belt would not be measured in this test, since they would have no effect on the leaf spring tension. Extrapolating to Vo from the curve, τ was determined from V_o_/e to be 12.0 seconds. This is less than the normal breathing frequency in premature infants (40–60/minute) [19], and indicates that minimal distortion of the tension gauge output in the form of integration of breathing motion will occur. Hence monitoring the average level of belt tension, the main purpose of the device, is not effected.

The viscoelastic properties of the electrode belt and Velcro connections were considered by wrapping the belt/tension gauge about a cylinder 22 cm in circumference. One hundred grams of tension was applied by tightening the strap; the response indicates a similar viscoelastic time constant to the previous test (Fig. 8, open circles). Thus the material properties of the electrode belt would not be expected to influence the clinical measurement beyond the initial decrease in the output of ~10%.

**Figure 9:**
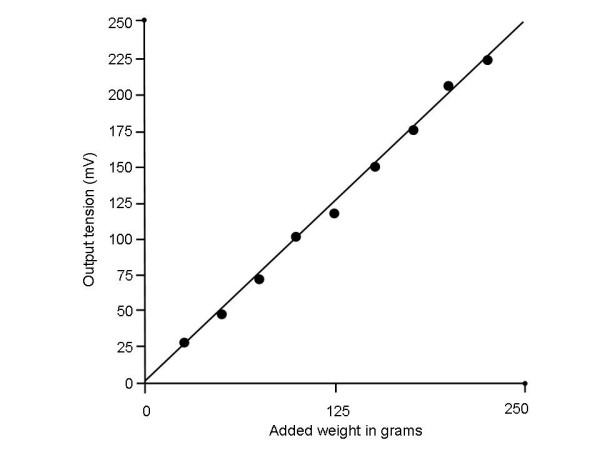
Measurement to determine the linearity of the device. Weights are added at 25 gm increments after the device was calibrated to 1 gm tension = 1 mV. The output was linear in the range from 25 g–225 g (r^2 ^= 0.99).

A static calibration curve was produced by recording the device output 2 seconds after releasing a weight in the weight range 25–225 gm and (Fig. 9). From linear regression analysis of the scatter plot points, r^2 ^= 0.99. Therefore the device exhibits highly linear behavior in the expected measurement range.

**Figure 10:**
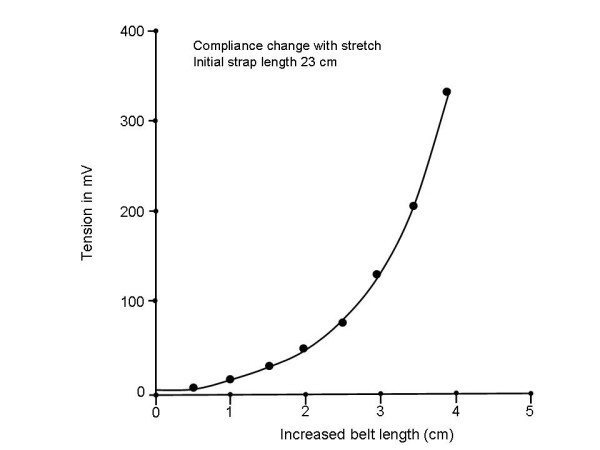
Compliance of the foam rubber electrode belt with increasing applied tension. Initially, belt length increases approximately linearly by several centimeters when up to 150 gm tension are applied. However, the belt begins to become inelastic at higher levels of tension applied. There is less than a 1 centimeter increase in belt length as applied tension goes from 200 gm to 300 gm. These values however, were above the clinically applied level of tension (<100 gm).

The compliance of the belt itself was nonlinear (Fig. 10). As tension was increased, the compliance decreased. Hence the electrode belt could not be used to accurately measure the magnitude of chest wall movements unless it is first calibrated to the specific level of tension and circumference of the patient. However, the direction of chest wall movement, and the relative movement, will be evident from the direction of the signal deflections.

The actual measured output was found to be 218 mV per 100 g tension (estimate was 230 mV – see Methods). However, for clinical use the output was adjusted through the calibration circuitry so that a 100 mV output reading corresponded to a 100 gm input.

### Clinical recordings

In the neonatal ICU, to obtain good contact between the ECG electrodes and the infant's skin, a belt tension of 30–90 grams was required as measured with the tension gauge. The subject used for testing the device was a 3 day old female, gestational age 25 weeks at birth, weight 526 g. Her breathing was assisted with a mechanical ventilator (35/min, PO_2 _= 34 mmHg) but otherwise her functions and vital signs were normal for the circumstance as confirmed by a physician. She was moving during the measurements and had the appearance of good health. After a level tension of 30–90 gm was applied as read from the gauge output, the reading slowly decreased by approximately 5 mV (5 gm tension) over several minutes, which was attributable in part to the viscoelastic property of the epoxy and also to redistribution of tension about the belt during patient movement. It was also noted that large-scale motion by the infant caused transient increases in tension of several tens of grams. However, the response of the device output to infant breathing was negligible. During the entire measurement period, the infant's heart rate was constant at 158 bpm at the applied levels of tension and the O_2 _saturation level was also constant at 89%. The respiratory parameters are provided in the accompanying table, Table 1 (the results of one subject).

**Table 1:** Relationship between Tension Level and Respiratory Parameters

Parameter (units)	30 g	50 g	90 g	full term
Resp frequency min^-1^	71.1 ± 3.7	75.7 ± 4.4	71.0 ± 4.1	60
Tidal volume ml/kg	6.0 ± 0.3	6.4 ± 0.4	7.8 ± 0.9	6–8
Min ventilation ml/min/kg	419 ± 20	480 ± 30	517 ± 41	400
Dyn compliance ml/cm H_2_O	.32 ± .02	.34 ± .02	.46 ± .07	1.00
Pul resistance cm H_2_O/L/s	186 ± 20	134 ± 11	185 ± 22	40
Work breathing g·cm/kg	24.1 ± 0.1	23.4 ± 0.2	36.9 ± 0.2	10–40

The clinical data showed some change in most respiratory functions during monitoring with three different levels of belt tension. In Table 1, respiratory frequency is higher than the rate for the normal term infant (60/min). The tidal volume increases slightly with increasing tension level but at all three levels it is in range for a normal term infant (6–8 ml/kg). Minute ventilation increased significantly with increased tension level, in tandem with the increase in tidal volume and the approximately flat respiratory frequency. The dynamic compliance also increase significantly with increased tension level, but was still far below the normal for a term infant (1.00 ml/cm H2O). Pulmonary resistance did not change significantly from 30–90 g of tension, but was still far above that of a normal term infant (40 cm H_2_O/L/s). There was a significant increase in the work of breathing from 30 g to 90 g tension. At all three levels work of breathing was in the normal range for a term infant (10–40 g • cm/kg).

## Discussion

In this study, the design and construction of an electrode belt tension gauge was described. The device was tested for mechanical and electrical response. It was then clinically tested using a preterm infant who was already connected to an electrode belt for monitoring the electrocardiogram in an ICU setting.

### Device properties

The tension gauge was constructed to minimize weight and encumbrance to the infant. The device was designed to convert the mechanical signal (electrode belt tension) to an electrical signal. Two strain gauges bonded to opposite sides of a leaf spring registered differing levels of resistance depending on the degree of tension imparted to the slide mechanism. During bench tests, the device exhibited a linear response to increasing tension levels within the range that would be expected during clinical measurement. The mechanical response to a step input was a second order function. The computed resonant frequency (400 Hz) was above the mechanical frequency range that would be expected to be encountered in a clinical setting.

For the creep tests, a constant load was applied during the test to allow for stiffness measurement [24]. The bonding epoxy did exhibit some creep (~10%) over the course of a few minutes when a constant level of tension was applied. However, the electrode belt was connected to the device frame, not to the leaf spring, and therefore its tension level would be unaffected by the properties of the bonding epoxy used to anchor the strain gauges. Yet, epoxy was also used to cement the Velcro tabs onto the device frame. Creep in this epoxy over time would be expected to diminish the actual tension in the electrode belt to which it was directly connected. This response would be anticipated to affect the measurement (decrease in tension reading over time). However, during actual clinical measurement, the decrease in tension over time was limited to about 10% at each tension level. Epoxies which exhibit less creep when subject to a constant stress should be used in subsequent manifestations of this measuring device [25]. One other difficulty was the nonlinear elastic response of the belt to differing levels of tension (Fig. 10). Thus with increased stretch, tension in the belt increased disproportionately. In terms of clinical recording, this would mean that there would be less compliance of the belt (greater force applied to the infant chest) for increases occurring at higher starting tension levels.

### Clinical result

The clinical table (Table 1) and other results suggest that differing levels of tension used to emplace the electrode belt had some effects on respiratory function in this infant. At intermediate belt tension level (50 g), improvement in respiratory function occurred. The intermediate tension level may have stabilized the infant's chest wall, which underwent paradoxical breathing [19], in a manner that promoted more efficient breathing. Premature infants are known to breath paradoxically during rapid eye movement (REM) sleep, due to the very high level of compliance of their chest walls. In the case of the intermediate tension level, the chest wall may be supported and stabilized in a manner that promotes more efficient breathing. However, it may also be the case that the infant's long-term breathing response will differ from the short-term results depicted in the table. Additional subjects and a longer monitoring time will be required to know if these results will hold for a representative population.

Even distribution of tension about the electrode belt was probably not achieved during clinical measurement. Areas with greater tension were likely located near the tension measuring device, where the belt was connected above the infant's chest. Lesser tension would be expected at the back, where the weight of the infant would partially prevent even distribution of the applied tension. Therefore, we would expect the degree of tension to be less than the read value at some areas about the circumference of the belt. Segments of the belt with reduced tension would not impart as much force on the torso and therefore would contribute less to the effect of the belt on respiratory function.

### Limitations and future directions

The piezoresistive transducers were bonded to a leaf spring with an epoxy that exhibited creep over several minutes time. This limited the accuracy of the measurement to determine whether electrode belt tension remained constant over time. Single recordings at varying levels of tension were made on one subject. Due to constraints in interaction with the patient, these recordings were done during a short period of time at one setting. It is uncertain whether the respiratory measurements at fixed levels of tension are time invariant. Supposing that these measurements are approximately time invariant, or that correction or normalization can be used to account for time-varying differences, a large population of subjects would still be required to determine the statistical significance of the effects of belt tension on respiratory function. Thus the approach that we have described is preliminary; other methodology may improve the accuracy of the measurements. Heart rate remained stable during the measurement interval; however, correlation of tension level and respiratory function to heart signals such as electrocardiogram and blood pressure would be useful to state more definitively whether the mechanical properties of the ECG belt affect the heart.

The instrument was not used to measure chest wall motion, but such information could improve understanding of the mechanism of paradoxical breathing. Although the tension gauge monitor was tested with an electrode belt attached to a premature infant, monitoring of belt tension would be useful in settings such as heart rate monitoring and variability [1-5], for sports and aerospace medical activities [6-9], as well as for electrical impedance tomography [10]. Monitoring of tension would assure that any change in respiratory or cardiovascular function is not due to the mechanical constraint offered by the belt. For very long term monitoring, the power supply of the device would need to be upgraded. Since some electrode belts and vests apply tension across the chest in multiple directions, monitoring of each axis will likely be important for understanding respiratory and cardiovascular effects.
